# Reducing the Risk of Cognitive Decline and Dementia: WHO Recommendations

**DOI:** 10.3389/fneur.2021.765584

**Published:** 2022-01-10

**Authors:** Neerja Chowdhary, Corrado Barbui, Kaarin J. Anstey, Miia Kivipelto, Mariagnese Barbera, Ruth Peters, Lidan Zheng, Jenni Kulmala, Ruth Stephen, Cleusa P. Ferri, Yves Joanette, Huali Wang, Adelina Comas-Herrera, Charles Alessi, Kusumadewi Suharya (Dy), Kibachio J. Mwangi, Ronald C. Petersen, Ayesha A. Motala, Shanthi Mendis, Dorairaj Prabhakaran, Ameenah Bibi Mia Sorefan, Amit Dias, Riadh Gouider, Suzana Shahar, Kimberly Ashby-Mitchell, Martin Prince, Tarun Dua

**Affiliations:** ^1^Department of Mental Health and Substance Use, World Health Organization, Geneva, Switzerland; ^2^Department of Neuroscience, Biomedicine and Movement Sciences, WHO Collaborating Centre for Research and Training in Mental Health and Service Evaluation, University of Verona, Verona, Italy; ^3^University of New South Wales, Sydney, NSW, Australia; ^4^Division of Clinical Geriatrics, Department of Neurobiology, Care Sciences and Society, Center for Alzheimer Research, Karolinska Institutet, Stockholm, Sweden; ^5^The Ageing Epidemiology Research Unit, Imperial College London, School of Public Health, London, United Kingdom; ^6^Institute of Public Health and Clinical Nutrition, University of Eastern Finland, Kuopio, Finland; ^7^Theme Inflammation and Aging, Karolinska University Hospital, Stockholm, Sweden; ^8^Department of Neurology, Institute of Clinical Medicine, University of Eastern Finland, Kuopio, Finland; ^9^Neuroscience Research Australia, Sydney, NSW, Australia; ^10^Population Health Unit, Finnish Institute for Health and Welfare, Helsinki, Finland; ^11^Faculty of Social Sciences (Health Sciences) and Gerontology Research Centre (GEREC), Tampere University, Tampere, Finland; ^12^Department of Psychiatry, Federal University of São Paulo, São Paulo, Brazil; ^13^Canadian Institutes of Health Research, Government of Canada, Ottawa, ON, Canada; ^14^Dementia Care and Research Center, Peking University Institute of Mental Health, Beijing, China; ^15^Beijing Dementia Key Laboratory, Beijing, China; ^16^Department of Health Policy, Care Policy and Evaluation Centre, London School of Economics and Political Science, London, United Kingdom; ^17^Public Health England, London, United Kingdom; ^18^Alzheimer's Disease International, Jakarta, Indonesia; ^19^Division of Non-communicable Diseases, Ministry of Health, Nairobi, Kenya; ^20^Mayo Clinic, Rochester, MN, United States; ^21^University of KwaZulu-Natal, Durban, South Africa; ^22^The Geneva Learning Foundation, Geneva, Switzerland; ^23^Public Health Foundation of India, New Delhi, India; ^24^Alzheimer Association, Quatre Bornes, Mauritius; ^25^Department of Preventive and Social Medicine, Goa Medical College, Goa, India; ^26^Faculty of Medicine, Clinical Investigation Centre Neurosciences and Mental Health Razi University Hospital, Tunis, Tunisia; ^27^Faculty of Medicine, University Tunis El Manar, Tunis, Tunisia; ^28^Centre for Healthy Aging and Wellness, Universiti Kebangsaan Malaysia, Kuala Lumpur, Malaysia; ^29^The Mona Ageing and Wellness Centre, University of the West Indies, Kingston, Jamaica; ^30^King's College London, London, United Kingdom

**Keywords:** dementia, dementia risk reduction guidelines, dementia risk reduction trials, WHO guidelines, cognitive decline

## Abstract

With population ageing worldwide, dementia poses one of the greatest global challenges for health and social care in the 21st century. In 2019, around 55 million people were affected by dementia, with the majority living in low- and middle-income countries. Dementia leads to increased costs for governments, communities, families and individuals. Dementia is overwhelming for the family and caregivers of the person with dementia, who are the cornerstone of care and support systems throughout the world. To assist countries in addressing the global burden of dementia, the World Health Organisation (WHO) developed the Global Action Plan on the Public Health Response to Dementia 2017–2025. It proposes actions to be taken by governments, civil society, and other global and regional partners across seven action areas, one of which is dementia risk reduction. This paper is based on WHO Guidelines on risk reduction of cognitive decline and dementia and presents recommendations on evidence-based, multisectoral interventions for reducing dementia risks, considerations for their implementation and policy actions. These global evidence-informed recommendations were developed by WHO, following a rigorous guideline development methodology and involved a panel of academicians and clinicians with multidisciplinary expertise and representing geographical diversity. The recommendations are considered under three broad headings: lifestyle and behaviour interventions, interventions for physical health conditions and specific interventions. By supporting health and social care professionals, particularly by improving their capacity to provide gender and culturally appropriate interventions to the general population, the risk of developing dementia can be potentially reduced, or its progression delayed.

## Introduction

With population ageing worldwide, dementia poses one of the greatest global challenges for health and social care in the 21st century. In 2019, around 55 million people were affected by dementia, with the majority living in low- and middle-income countries (LMIC). The total number of people with dementia is projected to reach 78 million in 2030 and 139 million in 2050 (1).

Dementia leads to increased costs for governments, communities, families and individuals. In 2019, the total global societal cost of dementia was estimated to be US$1.3 trillion, equivalent to 1.5% of global gross domestic product (GDP). This number is expected to more than double by 2030, reaching US$2.8 trillion (1).

Dementia can be overwhelming for families and caregivers, who are the cornerstone of care and support systems throughout the world. They are impacted by adverse psychological, physical, social and financial consequences (2). Furthermore, a lack of understanding about dementia leads to stigma and discrimination and people with dementia are frequently denied their human rights in both the community and care homes (3). Left unaddressed, dementia represents a significant barrier to global social and economic development.

Crucially, while chronological age is the strongest known risk factor for cognitive decline and dementia, dementia is not a natural or inevitable consequence of ageing. In fact, several studies have recently linked dementia to a number of potentially modifiable risk factors such as physical inactivity, tobacco use, unhealthy diets, and harmful alcohol use. Certain medical conditions–including hypertension, diabetes, hypercholesterolemia, obesity and depression–are also associated with an increased risk of dementia (4–6).

The increasing global threat that dementia poses, and the lack of established curative treatment make it important to focus attention on risk reduction. The concept of risk reduction incorporates both the factors that increase the likelihood of cognitive decline and impairment, as well as factors that contribute to neurocognitive resilience. Taken together, these measures can effectively reduce the risk of having to live with dementia and can contribute to diminishing the prevalence of dementia in the population. Additionally, by supporting health and social care professionals, particularly by improving their capacity to provide gender and culturally appropriate interventions to the general population, the risk of developing dementia can be potentially reduced, or its progression delayed.

To assist countries in addressing the global burden of dementia, the World Health Organisation (WHO) developed the Global Action Plan on the Public Health Response to Dementia 2017–2025, endorsed by the World Health Assembly in May 2017. It proposes actions to be taken by governments, civil society, and other global and regional partners across seven action areas, one of which is dementia risk reduction. This paper is based on the first WHO Guidelines on risk reduction of cognitive decline and dementia (7), and presents recommendations on evidence-based, multisectoral interventions for reducing dementia risks, considerations for their implementation and further research needs.

## WHO Methodology for the Development of Guidelines

The WHO guidelines on risk reduction of cognitive decline and dementia was developed by following a rigorous guideline development methodology that is described in the WHO Handbook for guideline development and uses the Grading of Recommendations Assessment, Development and Evaluation (GRADE) methodology (8, 9). To support the guideline development process, a steering group led by the Department of Mental Health and Substance Use at WHO was established and included representatives from WHO regional offices and relevant WHO departments and programmes. Two additional groups were established: a guideline development group (GDG) and an external review group (ERG). The GDG included a panel of academics and clinicians with multidisciplinary expertise on the conditions covered by the guidelines. The 12 risk factors and interventions presented in the guidelines were selected based on reports from two recent systematic reviews (10, 11) and those summarised in existing WHO guidelines, i.e., the Prevention and control of non-communicable diseases (12) and Integrated care for older people (ICOPE) guidelines (13). The choice of 12 risk factors was determined by their potential to be targeted for prevention of dementia, and/or the delay of the progression of cognitive decline. Existing relevant systematic reviews were identified for each of the 12 risk factors. The quality of existing reviews was assessed using the assessment of multiple systematic reviews (AMSTAR) checklist. A review of evidence was conducted for interventions related to the 12 risk factors of focus to evaluate their efficacy in reducing risk of cognitive decline and/or dementia for adults with normal cognition or mild cognitive impairment (MCI), and took into consideration the study design—randomised controlled trials (RCT) or observational studies, risk of bias, inconsistency, indirectness, imprecision and risk of reporting bias. Evidence was characterised as either high, moderate, low or very low.

The resulting WHO recommendations targeting nine (of the 12 reviewed) modifiable risk factors are based on evidence from 51 good-quality systematic reviews, including those of observational studies, published in the last 2 years and expanded by up to 5 years, when necessary. Further considerations taken into account included: the balance of benefits and harms of each intervention; values and preferences of the end-user involved; costs and resource use; acceptability of the intervention to health care providers; feasibility of implementation; and impact on equity and human rights. In addition to these systematic reviews, other related guidelines were considered in the development of the recommendations. Some of these are detailed in Box 1.

Box 1 **Related WHO guidelines and tools.**

- mhGAP Intervention guide for mental, neurological and substance use disorders in non-specialist health settings – version 2.0. Geneva: WHO; 2016. 173 p. Available from: http://www.who.int/mental_health/mhgap/mhGAP_intervention_guide_02/en/
- Prevention and control of non-communicable diseases: guidelines for primary health care in low-resource settings. Geneva: WHO; 2012. 72 p. Available from: http://apps.who.int/iris/bitstream/10665/76173/1/9789241548397_eng.pdf
- Package of essential non-communicable (PEN) disease interventions for primary health care in low-resource settings. Geneva: WHO; 2010. 85 p. Available from: https://www.who.int/publications/i/item/who-package-of-essential-noncommunicable-(pen)-disease-interventions-for-primary-health-care
- Global recommendations on physical activity for health. Geneva: WHO; 2010. 60 p. Available from: http://www.who.int/dietphysicalactivity/publications/9789241599979/en/
- Guidelines on Integrated care for older people (ICOPE). Geneva: WHO; 2017. 60 p. Available from: https://www.who.int/publications/i/item/9789241550109
- HEARTS Technical package for cardiovascular disease management in primary health care: evidence-based treatment protocols (2018). https://www.who.int/publications/i/item/hearts-technical-package [accessed August 25, 2021].
- Global age-friendly cities: a guide. Geneva: WHO; 2007. 82 p. Available from: https://www.who.int/ageing/publications/Global_age_friendly_cities_Guide_English.pdf
- Recommendations on healthy diet (2019). http://www.who.int/en/news-room/fact-sheets/detail/healthy-diet [accessed August 25, 2021].
- Strengthening health systems for treating tobacco dependence in primary care. Geneva: WHO; 2013. 78 p. Available from: https://www.who.int/publications/i/item/strengthening-health-systems-for-treating-tobacco-dependence-in-primary-care
- Framework on integrated people-centred health services. Geneva: WHO; 2016. 12 p. Available from: http://apps.who.int/gb/ebwha/pdf_files/WHA69/A69_39-en.pdf?ua=1

Taking into account these considerations, recommendations were classified by their strength (strong or conditional). Strong recommendations were issued when the multidisciplinary panel had clear confidence that the desirable effects of the intervention outweighed any undesirable effects. In case the panel was uncertain about the balance between the desirable and undesirable effects, a conditional recommendation was issued. Strong recommendations imply that most individuals would want the intervention and should receive it, while conditional recommendations imply that different choices may be appropriate for individual patients, and they may require assistance at arriving at management decisions. In some instances, even when the quality of evidence was low or very low, it was agreed that if the recommendation would be of general benefit, and this was seen to outweigh the harms, it may still be rated as strong.

The full evidence profiles underpinning the recommendations are available at the WHO website (https://www.who.int/publications/i/item/risk-reduction-of-cognitive-decline-and-dementia).

## WHO Recommendations on Risk Reduction of Cognitive Decline and Dementia

The guidelines are primarily intended for health care providers working at a first or second level facility or at district level, including basic outpatient and inpatient services. The health care providers may be doctors, nurses, or other cadres of health workers. In addition, the guidelines have implications for policy makers, and health care planners and program managers at national and international level. It is also crucial for the general population, which is more and more engaged in empowering itself and taking action, to maintain good cognitive health. This is especially true in a context where there has been a proliferation of consumer-directed solutions, many of them digital (app, mHealth), for which there is inadequate guidance offered as to their worth or effectiveness.

The recommendations are considered under three broad headings: lifestyle and behaviour interventions, interventions for physical health conditions and specific interventions (Table 1).

**Table 1:** WHO recommendations for risk reduction of cognitive decline and dementia.

Physical activity interventions	• Physical activity should be recommended to adults with normal cognition to reduce the risk of cognitive decline. • *Quality of evidence: Moderate* • *Strength of the recommendation: Strong* • Physical activity may be recommended to adults with mild cognitive impairment to reduce the risk of cognitive decline. • *Quality of evidence: Low* • *Strength of the recommendation: Conditional*
Tobacco cessation interventions	• Interventions for tobacco cessation should be offered to adults who use tobacco since they may reduce the risk of cognitive decline and dementia in addition to other health benefits. • *Quality of evidence: Low* • *Strength of the recommendation: Strong*
Nutritional interventions	• A Mediterranean-like diet may be recommended to adults with normal cognition and mild cognitive impairment to reduce the risk of cognitive decline and/or dementia. • *Quality of evidence: Moderate* • *Strength of the recommendation: Conditional* • Healthy, balanced diets should be recommended based on WHO recommendations on healthy diet. • *Quality of evidence: Low to high (for different dietary components)* • *Strength of the recommendation: Strong* • Vitamins B and E, polyunsaturated fatty acids, and multi-complex supplementation should not be recommended to reduce the risk of cognitive decline and/or dementia. • *Quality of evidence: Moderate* • *Strength of the recommendation: Strong*
Interventions for alcohol use disorder	• Interventions aimed at reducing or ceasing hazardous and harmful drinking should be offered to adults with normal cognition and mild cognitive impairment to reduce the risk of cognitive decline and/or dementia in addition to other health benefits. • *Quality of evidence: Moderate (for observational evidence)* • *Strength of the recommendation: Conditional*
Cognitive interventions	• Cognitive training may be offered to older adults with normal cognition and with mild cognitive impairment to reduce the risk of cognitive decline and/or dementia. • *Quality of evidence: Very low to low* • *Strength of the recommendation: Conditional*
Weight management	• Interventions for midlife overweight and/or obesity may be offered to reduce the risk of cognitive decline and/or dementia. • *Quality of evidence: Low to moderate* • *Strength of the recommendation: Conditional*
Management of hypertension	• Management of hypertension should be offered to adults with hypertension according to existing WHO guidelines. • *Quality of evidence: Low to high (for different interventions)* • *Strength of the recommendation: Strong* • Management of hypertension may be offered to adults with hypertension to reduce the risk of cognitive decline and/or dementia. • *Quality of evidence: Very low (in relation to dementia outcomes)* • *Strength of the recommendation: Conditional*
Management of diabetes mellitus	• The management of diabetes in the form of medications and/or lifestyle interventions should be offered to adults with diabetes according to existing WHO guidelines. • *Quality of evidence: Very low to moderate (for different interventions)* • *Strength of the recommendation:* S*trong* • The management of diabetes may be offered to adults with diabetes to reduce the risk of cognitive decline and/or dementia. • *Quality of evidence: Very low* • *Strength of the recommendation: Conditional*
Management of dyslipidaemia	• Management of dyslipidaemia at midlife may be offered to reduce the risk of cognitive decline and dementia. • *Quality of evidence: Low* • *Strength of the recommendation: Conditional*

## Discussion

### Guidelines' Implementation Considerations

Many of the interventions included in these guidelines are strongly related to managing risk factors for cardiovascular disease and diabetes. Therefore, the implementation of these recommendations should be combined, whenever possible, with the ongoing prevention programmes to reduce the risk of these conditions in the relevant target populations. For example, non-communicable disease prevention programmes need to include dementia as an integral part of action at global and national levels. Such an approach is particularly interesting as it provides opportunities to develop public health policies that will work in synergy and a single designed intervention has the capacity of impacting risk reduction efforts of more than one disease. Countries should include the recommendations in training and education programmes of healthcare providers, so that these interventions can be provided to all adults in primary health care. An important initiative aimed at leveraging mobile technologies to provide health information to those at risk of developing dementia is WHO's mDementia programme and its mDementia handbook (14). The handbook provides guidance on how to develop, integrate, implement and evaluate a national mHealth programmes and includes message libraries on dementia risk reduction. A summary of policy measures for effective dementia risk reduction is summarised in Box 2.

Box 2 **Policy actions for dementia risk reduction.**

- Link dementia risk reduction to existing non-communicable disease policies and programmes
- Integrate reducing the risk of cognitive decline and dementia into community services and social protection policies especially among older persons.
- Train the health workforce in early detection of cognitive decline and in providing interventions to reduce dementia risk
- Conduct campaigns (for example, through consumer-directed digital health) to educate and empower the general public about modifiable dementia risk factors and how these can be mitigated
- Integrate surveillance of cognitive decline and dementia in national demographic and health surveys to inform policy on prevention and treatment
- Use digital technologies and innovation to combat risks
- Invest in research to build the evidence base for risk reduction interventions and address research gaps
- Strengthen monitoring efforts to measure implementation of risk reduction strategies and their impact
- Strengthen collaboration between civil society, government, private sectors and institutions to achieve an impactful implementation of integrated risk reduction strategies

The optimal preventive effect may be obtained by addressing several risk factors at the same time. Collaboration among different stakeholders and multidisciplinary approaches will also be needed. Local adaptation is necessary to ensure that the guidelines are appropriate for the local conditions that affect the care of people with cognitive decline and dementia in health facilities and the community.

As countries consider how to implement these guidelines, the budgetary and human resource requirements, and other health systems implications should be analysed to identify what inputs and systems are currently available and what areas require additional investment. These may include training of health workers, supply of medicines and adaptations of health information systems to collect data on service utilisation.

### Limitations of the Process

While these guidelines used transparent and robust methodologies for evidence synthesis and the development of recommendations according to the WHO Handbook for Guideline Development, the limitations of the guidelines chiefly result from the inadequate evidence available for some of the research questions.

Though the prevalence of dementia is increasing in LMIC, most of the studies were conducted in high-income countries. Much of the evidence came from well-resourced settings, which could potentially impact on generalizability. The relatively short follow-up of most intervention trials limits our ability to judge the potential impact of the intervention on the development of cognitive decline and dementia, both of which have long prodromal periods. Additionally, most studies failed to distinguish between mid-life vs. late life risk factors. For example, the majority of the evidence relating to blood pressure-lowering is drawn from late life samples. However, the epidemiological evidence suggests that the association between high blood pressure and increased risk of cognitive decline or dementia is strongest for high blood pressure experienced in mid-life (e.g., 40–65 years) (15). Few studies distinguish between dementia subtypes (i.e., Alzheimer's, dementia with Lewy bodies, frontotemporal dementia, vascular dementia) with most studies only reporting on all-cause dementia.

Few data were available for interventions targeting some of the modifiable risk factors and reporting outcomes related to dementia or cognitive decline. Therefore, although social isolation, depression and hearing loss were included in the initial scoping, no recommendation could be made relating to these risk factors. However, new evidence is emerging for interventions targeting these risk factors and will be considered when the guidelines are updated.

In instances where the evidence was limited, we took advantage of the GRADE methodology (9, 16), which recognises that, in addition to the evidence base, other aspects that are expected to inform the recommendations. These include consideration of values such as gender equality, equity and the protection of human rights, feasibility, resource use and the knowledge and experience of the Guideline Development Group experts.

These limitations, together with the critical analysis of the evidence collected, which is presented in the current issue in a separate paper (17), emphasise the need for further research to fill key knowledge gaps in ways that can enable improved future guidance.

Major research questions that need to be addressed as a priority are listed in Box 3, and complement the conclusions drawn from the paper published in another issue of this journal (17).

Box 3 **Key areas for further research.**

- Explore single and multi-domain approaches addressing multiple risk factors for primary and secondary prevention of dementias based on evidence on risk/protective factors and the relationship with other chronic diseases
- Understand the influence and interactions of non-modifiable (e.g., gender, genetics, age) and modifiable (e.g., physical activity, diet, and cognitive stimulation) risk and protective factors for dementia in population-based samples
- Determine the feasibility, optimal mode of administration, and effectiveness of interventions to address risk factors for dementia, including identifying and managing barriers to, and facilitators of, implementing evidence-based guidance and policy recommendations
- Study the effectiveness of eHealth applications targeting risk reduction and behaviour change messaging

## Conclusion

In this article we propose evidence-based recommendations to reduce the risk of cognitive decline and dementia and will facilitate the implementation of WHO's Global Action Plan on the Public Health Response to Dementia 2017–2025, specifically to achieve the global target for dementia risk reduction.

The recommendations are designed to inform policymakers, healthcare providers, as well as other stakeholders. Given that the risk factors identified by the studies and recommendations proposed in the guidelines are shared among other diseases, especially with cardiovascular diseases and diabetes, it is of particular importance to identify how to strategically develop interventions and/or integrate such recommendations in a manner that will maximise the impact and increase their cost efficiency.

New evidence in these areas are regularly monitored by WHO, in consultation with international experts and academic partners. These guidelines will be reviewed in around 5 years, unless an earlier review and update is warranted by breakthrough research. We also expect revisions to include relevant new questions or areas currently not covered by these guidelines.

We intend that the publication of the WHO guidelines, and the evidence-base that underpins them, will stimulate interest in the global scientific, policy and practitioner communities and thereby contribute to increase research capacity and strengthen evidence from countries/regions where needed. Though comprehensive and quality evidence has been gathered in the last decade on risk factors for dementia and cognitive decline, efforts should be targeted at improving the representation of LMICs. To achieve that, global prioritisation and coordination are necessary to ensure that ongoing dementia research covers critical areas within dementia risk reduction in ways that harmonise investigative approaches and reduce redundancies. Moreover, research efforts must be rooted in equity, diversity, and inclusiveness, be person-centred and family inclusive. This also necessitates building greater research capacity in all income settings and coordinated actions at the global level with all stakeholders.

## Data Availability Statement

The original contributions presented in the study are included in the article/supplementary material, further inquiries can be directed to the corresponding author.

## Author Contributions

TD and NC contribute to overall coordination of the guideline development. NC wrote the first draft of the manuscript. All authors contributed to manuscript revision, read, and approved the submitted version.

## Funding

KA is funded by NHMRC Fellowship 1102694. RP and LZ are funded by the NHMRC Dementia Centre for Research Collaboration. LZ is also funded by NHMRC Grant 1100579. MK is funded by Academy of Finland [Grants Nos. 317465 and 335524], the European Union Joint Programme – Neurodegenerative Disease (JPND) project EURO-FINGERS [Academy of Finland Grant No. 334804], Stiftelsen Stockholms Sjukhem, Center for Innovative Medicine (CIMED) at Karolinska Institutet, Knut and Alice Wallenberg Foundation, the Swedish Research Council for Health, Working Life and Welfare, Region Stockholm grants (ALF, NSV), and Konung Gustaf V:s och Drottning Victorias Frimurarstiftelse. AB and MB are funded by the European Research Council (ERC) [Grant No. 804371]. AB and RS are funded by the Finnish Cultural Foundatio. AB is also funded by the Academy of Finland [Grants Nos. 287490 and 319318] and Alzheimerfonden.

## Author Disclaimer

The authors alone are responsible for the views expressed in this article and they do not necessarily represent the views, decisions or policies of the institutions with which they are affiliated.

## Conflict of Interest

The authors declare that the research was conducted in the absence of any commercial or financial relationships that could be construed as a potential conflict of interest.

## Publisher's Note

All claims expressed in this article are solely those of the authors and do not necessarily represent those of their affiliated organizations, or those of the publisher, the editors and the reviewers. Any product that may be evaluated in this article, or claim that may be made by its manufacturer, is not guaranteed or endorsed by the publisher.
